# Internet use and health: Connecting secondary data through spatial microsimulation

**DOI:** 10.1177/2055207616666588

**Published:** 2016-09-02

**Authors:** Ulrike Deetjen, John A Powell

**Affiliations:** 1 Oxford Internet Institute, University of Oxford, UK; 2 Nuffield Department of Primary Care Health Sciences, University of Oxford, UK

**Keywords:** Internet, eHealth, health services, spatial microsimulation, secondary data, data linkage, big data

## Abstract

**Objective:**

Internet use may affect health and health service use, and is seen as a potential lever for empowering patients, levelling inequalities and managing costs in the health system. However, supporting evidence is scant, partially due to a lack of data to investigate the relationship on a larger scale. This paper presents an approach for connecting existing datasets to generate new insights.

**Methods:**

Spatial microsimulation offers a way to combine a random sample survey on Internet use with aggregate census data and other routine data from the health system based on small geographic areas to examine the relationship between Internet use, perceived health and health service use. While health research has primarily used spatial microsimulation to estimate the geographic distribution of a certain phenomenon, this research highlights this simulation technique as a way to link datasets for joint analysis, with location as the connecting element.

**Results:**

Internet use is associated with higher perceived health and lower health service use independently of whether Internet use was conceptualised in terms of access, support or usage, and controlling for sociodemographic covariates. Internal validation confirms that differences between actual and simulated data are small; external validation shows that the simulated dataset is a good reﬂection of the real world.

**Conclusion:**

Spatial microsimulation helps to generate new insights through linking secondary data in a privacy-preserving and cost-effective way. This allows for better understanding the relationship between Internet use and health, enabling theoretical insights and practical implications for policy with insights down to the local level.

## Background

Internet use carries the promise of health benefits: through supporting informed decision-making and self-care,^1–3^ improving interactions with health professionals,^4–6^ or providing online social support.^7–9^ Online resources may also help to reduce unnecessary health service use,^7^ reduce emergency room visits^10^ or increase health service use.^11–13^ Moreover, Internet use may influence how individuals perceive or rate their health.^7,9,14^ However, using the Internet may also have no, or negative, effects, such as confusion due to information overload, anxieties and unrealistic expectations due to exaggerations online, or lower satisfaction with one’s subjective wellbeing for various reasons,^15–17^ partially depending on how it is used and by whom.^16,18–20^

With health systems under constant pressure to save money and improve quality, there are hopes for the Internet to enhance cost-effectiveness and service quality through new digital services which support a model of patient-centred care.^21^ For society more generally, the Internet also has the potential to reduce health inequalities by reducing some of the barriers to accessing traditional care, especially for those with stigmatised conditions.^11^ In England, the National Health Service (NHS) has commissioned the project ‘Widening Digital Participation’ in 2013. This multi-million pound programme conducted by the Tinder Foundation aims to increase the share of Internet users especially among marginalised populations, and educate them about using online health resources.^22^ Similarly, the UK Government Digital Inclusion Strategy proposes improved health as one of the motivations of bridging the digital divide.^23^

However, large-scale evidence on the health effects of Internet use is still scant.^24^ Previous work has provided valuable insights into the association between Internet use and health-related outcomes for specific conditions^7–14^ but, to our knowledge, no large-scale studies of the overall relationship between Internet use, perceived health and health service use exist. Better understanding of this relationship is relevant from a theoretical perspective, as it helps to connect the literature on effects of Internet use^25,26^ with existing models of health service use^27–31^ and perceived health^32,33^ that partially predate the emergence of the Internet. A theoretical discussion of how these models are connected is available elsewhere.^34,35^ This research is also relevant from a practical perspective, as both digital inclusion, improving population health and making the health system more efficient are high on policymakers’ agendas around the world, as explained for the context of England above. At the same time, there are surveys on Internet use across the world as summarised in the World Internet Project,^36^ as well as vast amounts of routine data in health systems that have not yet been exploited to consider the relationship of digital participation and health.

In this article, we propose spatial microsimulation as a way to combine existing datasets in order to derive new insights. In spatial microsimulation, a simulated dataset based on probabilistic methods is created from existing secondary data (existing surveys and routine data from the health system). As a spatial model, this provides a simulated dataset of all individuals in a given geographic area with their individual (simulated) health service use and Internet use data, derived from routine sources (which do not necessarily have full population data, but which can be tied to the spatial location). After careful internal and external validation of the resulting dataset, and with some analytical restrictions that will be explained in this article, the simulated data can then be used to examine associations between characteristics of individuals in that geographic area.

Based on this simulated dataset, this research examines associations between Internet and health for three user concepts: how individuals access the Internet (access user concept), how they are supported and in need of support (support user concept), and whether they use the Internet for health-related purposes (usage user concept). These user concepts are derived from the digital divide literature: the access divide as the difference between those with and without access to the Internet and devices to access it;^37^ the support divide in terms of the availability of proxies or other sources of help both for technical questions and for finding and interpreting information;^1,38,39^ and the usage divide which relates to what people are doing and are able to do online.^40,41^ Table 1 presents an overview of all three Internet user conceptualisations in this research.

**Table 1.** Internet user conceptualisations in this research.

	Internet user concept		
	*Access*	*Support*	*Usage*
**User** (79%)	Next-generation user (54%)	Independent user (36%)	Health user (55%)
	*User with access on mobile and/or several devices*	*User who worked things out without help*	*User who seeks health information online*
	First-generation user (25%)	Supported user (26%)	Non-health user (24%)
	*All other users with access (e.g. On a laptop or desktop)*	*User who regularly receives others’ help*	*User who does not seek health information online*
		*Unsupported user (17%)*	
		*user who did not work out things and received no help*	
**Non-user** (21%)	Ex-user (3%)	Proxy access (4%)	
	*Non-user who used the Internet in the past*	*Non-user who asked somebody for Internet use*	
	Never-user (18%)	Proxy availability (11%)	
	*Non-user who never used the Internet*	*Non-user who knows someone with Internet access, but has not asked*	
		Fully excluded (6%)	
		*Non-user with nobody to use the Internet for them*	

Access is conceptualised based on Dutton and Blank,^36^ support and usage conceptualisation by authors. All percentages refer to the population of England based on the Oxford Internet Surveys (OxIS) used as one of the datasets in this research (see data section).

The benefit of the chosen spatial microsimulation approach is that no additional quantitative data collection is necessary (and may not be possible), thereby offering an efficient way to create new insights from existing data. In addition, one of the barriers to the secondary use of health datasets has been the concern over privacy and confidentiality with respect to individual health records, and this contributes to the challenges in linking health data with data from other sources at an individual level. As a simulated dataset, this is a privacy-preserving way of generating microdata for research.^42^ While a simulated dataset based on probabilistic methods will not be as accurate as obtaining measures from everyone in the population, it provides a useful basis to explore connections between Internet use, health and health service use. Of course, controlling for the known demographic and socio-economic correlates of Internet non-use, which at the same time are determinants of health more generally, is essential.^43^ Spatial microsimulation has been used in other health-related contexts, such as analysing implications of social media use^44^ and evaluating the factors associated with access to GP services,^45^ and in specific health conditions such as depression^46,47^ and estimating smoking prevalence.^48,49^ The purpose in these studies was estimating the geographic distribution of a certain variable of interest and using it for some subsequent analyses, whereas our study primarily employs spatial microsimulation as a technique for linking datasets, using geographical distribution as the connecting element rather than an end in itself. This research demonstrates the usefulness of spatial microsimulation to research the relationship between Internet and health through existing secondary data, enriches the existing health-related spatial microsimulation work with its focus on Internet use as more recent area of interest in health research, and provides large-scale insights on all of England combined with insights that can be broken down to the local level. This is useful as both Internet use and health differ between different areas.^50,51^ For example, digital exclusion is highest in England’s rural areas bordering Scotland and Wales, and lowest in and around London.^52^ Similarly, general health declines from south to north,^53^ and differs between smaller areas. Travelling east from Westminster in London, every two tube stations represent one year of life-expectancy lost; this is known as the ‘Jubilee line of health inequality’.^54^ England is a good case example for research into the influence of the Internet on health and health service use using a spatial microsimulation approach. In addition to the relevant policy context described above, England has a population with universal access to healthcare via a centralised health service (the NHS), an important feature for analysing effects on health service use as there might be many other factors which prevent individuals from seeking medical care (for example, low income, lack of insurance coverage etc.). These factors have been included in theoretical models on health service use,^27^ and acknowledged as a major limitation in US-based research on the Internet’s effects on health service utilisation.^13^ In addition, data is available on NHS use, and there is substantially wider computerised data capture and exchange than, for example, in the USA, Canada or Germany.^55^ Indeed, the UK government is at the forefront of the open data movement.^56^ There is a national census conducted every 10 years by the Office of National Statistics (ONS), which contains information on perceived health and long-term health conditions, and there is a nationally representative random sample longitudinal survey on Internet use.^57^ All of these datasets will be presented in more detail in the next section.

## Data

This research used three data sources that were combined by spatial microsimulation: The Oxford Internet Surveys (OxIS), the English census and Hospital Episode Statistics (HES). This connection is necessary as no single dataset exists which would allow us to research this relationship. Potentially eligible datasets, such as the British Household Panel Survey (BHPS)/UK Household Longitudinal Survey (UKHLS), the Opinions and Lifestyle Survey (OLS), and the Adult Media Use (AMU) survey by the Office of Communications (OfCom),^58–60^ have very limited detail on Internet use and its antecedent factors, or do not include the relevant health outcome constructs. However, these datasets are still valuable resources for external validation of the simulated dataset, which will also be described in this paper.

The **OxIS** provides a biannual random sample survey that has been conducted offline in a two-stage sampling process since 2003: a diverse set of 260 output areas (OAs) in terms of their area classification, urban/rural distinctions and regions in England were selected first, then around 10 individuals within each OA were surveyed. It contains fine-grained information on 2,150 individuals in England (in 2013) about what individuals do online, their attitudes about a wide range of Internet-related issues, their skills, as well as information about their offline context. As the interviews take place offline using a traditional paper-and-pen method, OxIS includes all kinds of users and non-users of the Internet, both from households and a range of communal establishments such as estates for the elderly.^36^ The data is released publicly upon request, although the publicly released version does not contain the sensitive elements of OAs (which are used for the simulation and validation of the resulting dataset in this research).

The **census** is a count of the population conducted every 10 years by the ONS, which contains aggregated information on perceived health, long-term health conditions, and the most important sociodemographic features. For all 171,372 OAs in England, it contains the number of individuals in given categories of gender, age, education, socioeconomic status (National Statistics Socio-Economic Classification (NS-SeC)) and long-term health conditions, as well as how individuals rated their health on a scale from 1–5. The outcome variable used in this research was created by calculating the average health rating per OA for the specific gender, age, education, socioeconomic status (NS-SeC) and presence of long-term conditions of the individual.

Finally, **HES** contains routinely collected aggregated data on hospital inpatient stays (number of unique individuals who went to hospital each month) as a measure of health service use, aggregated based on OAs, gender, and age groups as released for this research.^61^ The outcome variable used for this research was formed by dividing the number of unique individuals in hospital each month by the number of individuals in the same age/gender category in the OA, so that the resulting variable ranges from 0–12 (if everyone in the OA in the age/gender group was hospitalised at least once every single month). As HES data is automatically created procedural data, it does not contain any self-reported patient measures. The dataset was obtained from the Health and Social Care Information Centre (HSCIC) following an application process which evaluated the available security measures as well as the use to which the data was being put in this research.

**Figure 1.**
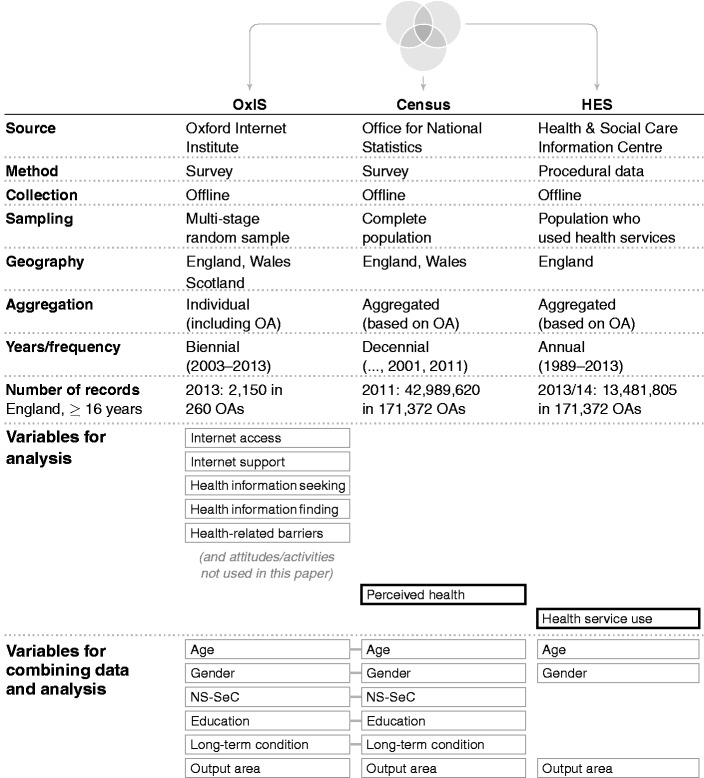
Available datasets. Oxford Internet Surveys (OxIS) and the census are connected through spatial microsimulation. Hospital Episode Statistics (HES) data is tied to the resulting simulated dataset based on geographic location (output area), which is available in all three datasets. NS-SeC: National Statistics Socio-Economic Classification; OA: output area.

All datasets contain OAs, which form the common basis for combining the datasets. OAs are small areas that consist of around 300 individuals, built from clusters of adjacent postcode units. They are formed based on the census data using geographic and socio-economic characteristics (such as tenure of household and dwelling type) to make them as socially homogeneous as possible.^62^ As mentioned above, they exist in OxIS for every respondent due to being the first stage of the sampling process and constituting the aggregation level for both census and HES data. Figure 1 provides an overview of the three datasets, their most important characteristics in terms of data collection, geographic coverage and volume, as well as the variables used for the spatial microsimulation process and data analysis.

## Methods

In short, the simulation worked as follows: for every OA – for which the aggregate characteristics of gender, age, education, socioeconomic status and long-term conditions are known from the census – the spatial microsimulation algorithm determined which of the individuals from the survey (OxIS) need to be chosen in order to best replicate who lives in the respective OA, so that the resulting dataset is a simulated dataset with 42,989,620 individuals (everyone in England over the age of 16 years) with their Internet use, attitudes and skills (from OxIS), perceived health and long-term health conditions (from the census) and health service use (from HES).

### Choice of constraint variables

Selecting the most suitable individuals for an OA to create the simulated dataset relies on so-called constraint variables, which must fulfil several conditions. Firstly, they need to exist in both the census and the survey dataset sharing the same definition and categories. Secondly, the constraint variables must be correlated with the variables taken to the small area level (Internet use, and the different user concepts derived from the digital divide literature in this case). Finally, they also need to be correlated to the outcome variables of interest (health and health service use in this case).^63,64^

Fulfilling these conditions, the proposed constraint variables in this research were age, education, existence of a long-term health condition, gender, socio-economic status (operationalised by the NS-SeC) and locality (in terms of area classification, urban/rural distinctions and different regions within England) in order of decreasing importance, as inferred based on a logistic regression on Internet use from the individual survey data, and Goodman-Kruskal gamma and chi-square tests on the aggregate census data. The order of the constraint variables is relevant, as the chosen iterative proportional fitting (IPF) algorithm matches the survey with the census data more closely with each constraint variable, so that variables deemed analytically more important should be entered last.^64^

Locality as a constraint variable is one of the special features of this research, as it is often lacking from the detailed individual-level dataset. Being able to include locality (the area characteristics of the specific OA) as a constraint was important as both health and health-related Internet use differ by location.^65,66^ In addition, while areas may consist of similar individuals, they may have different contextual features, so that geodemographic factors should be included in the model.^67^ Finally, factoring in locality also responds to the need to enable more local specificity of the dataset,^68^ particularly as health and Internet use differ across areas.^50,51^ The inclusion was possible as OxIS, unusually, includes the OA of each respondent, similarly to a few selected other models in the literature.^63,69^ Finally, age and education were used as cross-tabulated constraint variables. Being able to do this is one of the advantages of spatial microsimulation,^70^ and is useful as the population’s formal education levels have increased across cohorts between the middle of the 20th century and today.^71^ Ethnicity was considered as a constraint variable, but had no significant effect on either Internet use or health. This may be attributed to the different historic context of ethnicity in the UK (compared to the USA) and is in line with other UK research on Internet use.^72^

### Linkage of the datasets

With the chosen constraint variables, the population of Internet users for each OA were determined through reweighting the random sample survey so that it fitted small area population statistics.^73^ This research employed the IPF algorithm to do so, an algorithmic approach to estimating which individuals of the survey dataset are most suitable for replicating the actual population in the OA. IPF is only one of the possible approaches to spatial microsimulation, although most available approaches result in relatively similar results.^68^ IPF has the benefits of being relatively straightforward to apply and explain, and being widely used, avoiding local optima of the solution and guaranteeing convergence of the solution, as well as efficient use of computational resources.^74,75^ Good overviews of the different possible approaches of spatial microsimulation are provided in several other studies.^68,76^

**Figure 2.**
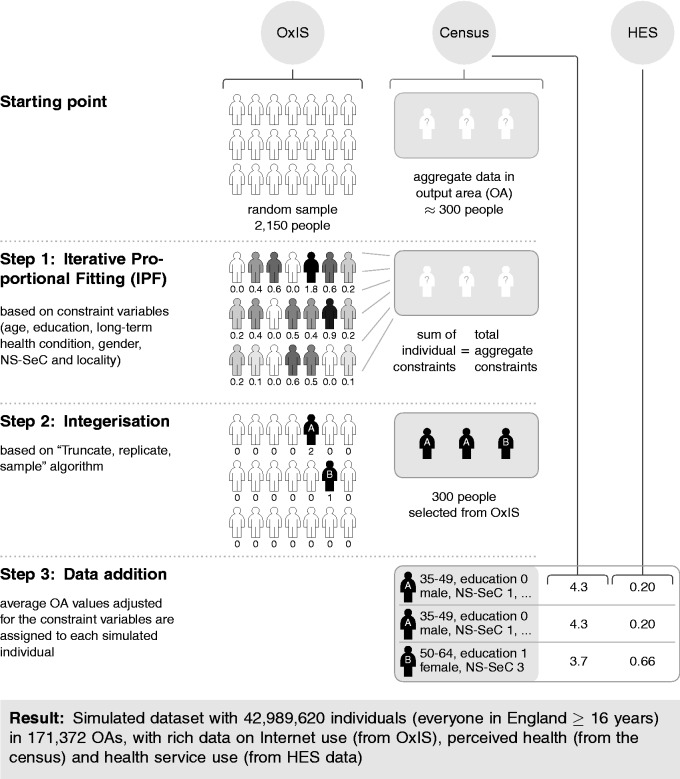
Spatial microsimulation process. A three-step process (example shown for one output area (OA) only) combines the datasets to analyse Internet use, health and health service use. Numbers are for illustration purposes only. HES: Hospital Episode Statistics; NS-SeC: National Statistics Socio-Economic Classification; OxIS: Oxford Internet Surveys.

In IPF, observations were weighted for each OA until the sums of the individual counts of the chosen constraint variables converged towards the totals for each constraint variable in the OA, with 10 iterations as a compromise between speed and accuracy of the results.^77^ The disadvantage of IPF is that it produces non-integer weights, as opposed to other combinatorial optimisation approaches such as simulated annealing.^75^ In order to improve interpretability of the results and reduce the size of the resulting dataset, the resulting weights were then integerised to analyse ‘whole individuals’. In the final step, the health outcome concepts from the census and HES were added (see Figure 2).

As an example, consider a hypothetical OA with 300 individuals. With the initial starting point of 2,150 individuals from OxIS, the first step consisted of determining how often each individual must be selected to recreate the population in the example OA. For example, if 160 of the 300 individuals are female (based on the census information), then the sum of the weights for all 2,150 females for that OA should be 160. Similarly, if there are 12 females above the age of 85 years in the OA, then the sum of all individual weights should add up to that number – and so on for education, NS-SeC and locality. Of course, based on the survey data available, not all constraints may be perfectly fulfilled at the same time, which is why IPF iteratively determines the best weights to match the population counts for each of the constraint variables as closely as possible. Good overviews of the mechanics of IPF are and their implementation in R are available in the literature,^74,78^ with further examples of IPF being provided in several research studies.^48,64,79,80^

Inevitably, the result of the reweighting process results in fractions such as 1.89 of individual A, 0.76 of individual B etc. so that it is useful to integerise the weights to obtain interpretable results and reduce the size of the resulting dataset. This research uses TRS (‘truncate-replicate-sample’), which is one of the integerisation algorithms that combines probabilistic elements of selecting individuals, while still ensuring that each individual with a weight of larger than one is selected at least once into the example OA.^75^ While taking up more computational resources compared to other approaches (such as simple rounding or proportional probabilities), and potentially not being applicable in situations where probabilistic effects are not desired, TRS created the smallest errors regardless of which validation measure was evaluated^75^ (as will be discussed in the next section on internal validation). Due to the probabilistic nature of integerisation, the internal validation section presents average values after 10 simulations.

After integerisation, the interim result is a simulated dataset with everyone in England over 16 years and how they use the Internet. Still missing is the third step of adding the health-related data from the census and HES data: Each individual in the simulated dataset was assigned the average perceived health, long-term health conditions and the average inpatient and outpatient visits to hospital. Crucial here is that not only the average for all 300 individuals in our example OA are used, but the values adjusted for the constraint variables such as gender, age, long-term health conditions, education and NS-SeC for the specific OA obtained from cross-tabulated census tables, so that for example, a 69-year-old women with a health condition and low NS-SeC is assigned the average value for women between 65–74 years with long-term health conditions and low NS-SeC in the specific OA. Ecological inference (creating individual level data from aggregate area-level data) is usually complicated,^81^ though it is more straightforward here as the census data is available in cross-tabulated form. This allows for reasonably approximating an average individual level of perceived health for an individual in a certain area, especially given that these areas are formed based on similar socio-economic characteristics.

After repeating these steps for all the other 171,372 OAs, the final result of the spatial microsimulation process is a simulated dataset with 42,989,620 individuals (everyone in England over the age of 16), which now allows for the joint examination of associations between Internet use, health and health service use, after internal and external validation checks are completed.

### Internal validation of the simulated data

Statistical checks were undertaken to determine internal validity for the spatial microsimulation process based on all 171,372 OAs in England. Long-term health conditions amounted to only 147,884 OAs, as those OAs for which the total sum of all long-term health condition categories did not match the total number of individuals in the census data source files were excluded from the calculation of internal validation measures. In addition, reliability checks were done to see to what extent OxIS respondents are replicated in those simulated areas where the original respondents actually came from.

For internal validation, Pearson’s R was used as a first indicator for goodness of fit, to evaluate how well the simulated totals for each of the constraints correlate with the actual totals. The average value of Pearson’s R obtained based on 10 simulations across all constraints is 0.98 (Locality: 1.00, NS-SeC: 0.98, Gender: 0.95, Long-term health condition: 0.99, Education: 0.97, Age: 0.99). It is not uncommon to reach values near one in constraint evaluation, with 0.9 as the recommended threshold for acceptance.^78^ Similarly, the Chi-square test was used to examine the differences between the actual and the simulated data. This gave a *p*-value = 1 across all constraints and for each constraint separately after removal of all areas in which any of the cell values were smaller than five. The result confirms that differences between the actual and the simulated data are very small and based on chance only.

The standardised absolute error (SAE) was used to evaluate the fit between actual and simulated totals for each area relative to the number of observations (individuals above the age of 16 years in England)^82^ and was 17.0% in this research. While there is no uniformly accepted threshold, the SAE should be below 20% in about 80% of the areas^83^ which is precisely the case in this research. In addition, the root mean squared error (RMSE), which rests upon the assumption that small differences are less problematic and hence penalises larger deviations, is 30.7, similar to previously reported values.^84^ The implications of the SAE and RMSE are more easily understood by comparing the actual proportions (based on the census) and the simulated proportions (based on the aggregated counts of the simulated dataset).^77^ Table 2 shows that the aggregate differences between both for each of the individual-level constraint variables are relatively small. While ethnicity was not included as a constraint variable due to not having a significant relationship with the target variables of interest, deviations between actual and simulated values were relatively small as well (actual/simulated: Asian – 4.9%/5.2%; Black – 1.4%/2.3%; White – 91.8%/90.9%; Other – 1.8%/1.5%).

**Table 2.** Internal validation results for individual constraint variables.

Proportion of individuals		Simulated	Actual
*Age, years*	*16–24*	13.8%	13.9%
	*25–34*	15.2%	15.3%
	*35–49*	26.7%	26.8%
	*50–64*	22.7%	22.9%
	*65–74*	11.0%	10.9%
	*75–84*	7.3%	7.2%
	*85+*	3.1%	3.1%
*Education*	*No qualification*	19.3%	19.1%
	*Level 1*	18.5%	18.7%
	*Level 2*	19.3%	19.5%
	*Level 3*	13.1%	12.8%
	*Level 4*	29.7%	29.9%
*Long-term health condition*	*Yes*	17.8%	17.9%
	*No*	82.2%	82.1%
*Gender*	*Male*	48.3%	48.6%
	*Female*	51.7%	51.4%
*NS-SeC*	*Class 1*	35.2%	35.3%
	*Class 2*	24.1%	24.2%
	*Class 3*	29.3%	29.0%
	*Unemployed*	4.1%	4.1%
	*Students*	7.3%	7.5%

Differences between the totals of the simulated dataset and the actual totals (based on the census) are very small. The numbers are based on the example region ‘South East’ in England.

As a final step in internal validation it was useful to check how many individuals are replicated into those areas where they are actually taken from in the survey data, which is possible in this case as OxIS records the OA of every survey participant. On average (based on 10 consecutive simulations), the simulated dataset replicates about 73% of individuals into their original areas. Aggregating these 10 simulations, the proportion of individuals replicated into their areas asymptotically approached 100%, reaching 97% based on the 10th simulation.

### External validation with comparable datasets

External validation involved the comparison of findings from the simulation with findings from other datasets. This is often difficult due to a lack of suitable datasets.^68^ In this case, there were three datasets available for external validation: The Internet access module of the OLS, which is a survey by conducted face-to-face by the ONS, the AMU conducted by the UK communications regulator OfCom, and ‘Understanding Society’, the UKHLS.^58–60^ All of these are not as fine-grained as OxIS in terms of how individuals access the Internet, but asked for data on Internet use, and health-related Internet use and/or perceived health, and hence provided a useful comparison of the overall proportion of individuals in each category (see Table 3).

**Table 3.** External validation of overall proportions.

		OxIS	Simulated	OLS	AMU	UKHLS
*Internet users*		79%	79%	85%	78%	83%
*Health information seekers*	*Monthly*	33%	31%	44%	31%	–
	*Weekly*	13%	11%	–	10%	–

AMU: Adult Media Use; OLS: Opinions and Lifestyle Survey; OxIS: Oxford Internet Surveys; UKHLS: UK Household Longitudinal Survey.

Differences for selected key items are relatively similar across datasets. The OLS and the UKHLS project a higher number of Internet users and health information seekers overall, as OxIS asked whether individuals ‘personally use the Internet on whatever device at home, work, school, college or elsewhere'. In contrast, AMU listed all devices (personal computer, laptop, netbook or alternative device), but restricted the question to the home, while the OLS and the UKHLS asked for general use without specifying devices or locations. In addition, OLS asked for ‘Health information seeking in the last three months’, whereas OxIS (and the simulated dataset) and AMU ask for whether individuals ‘search for health information at least monthly’.

Additionally, the OLS and the UKHLS feature a question on how individuals would rate their health status, which is one of the target variables of the spatial microsimulation model. This enables the relationship between Internet use and perceived health status to be researched on the same dataset, and thereby to validate the simulated dataset on this level as well. Table 4 summarises the average perceived health across user groups. The relationship also holds true when looking at the numbers separated by age and gender, though without significant differences in the means of users versus non-users for the lower two age groups (18–24 and 25–34 years).

**Table 4.** External validation of relationship between Internet use and average health.

	Simulated	OLS	UKHLS
**Users**	2.79	2.81	2.80
**Non-users**	2.55	2.36	2.40

OLS: Opinions and Lifestyle Survey; UKHLS: UK Household Longitudinal Survey.

The table shows the average perceived health (scale 1–3, 3 being the highest). Non-users are of lower health than users in both the simulated dataset and the two external validation datasets that contained data on perceived health. The overall lower health for non-users in the external validation datasets (2.36 in the OLS and 2.40 in the UKHLS compared to 2.55 in the simulated dataset) can be attributed to the circumstance that the survey featured a higher proportion of people with long-term health conditions. While OxIS, which the simulated dataset is based upon, only had 16% of individuals with a long-term health condition, both the OLS and the UKHLS included 35%. For comparison, based on the census, about 21% of individuals in England suffer from a long-term health condition.

### Data analysis

Standard analytical approaches can be applied to the analysis of simulated data, such as multiple regression or structural equation modelling as in this paper. However, *p*-values as indicators of significance that sample results are also true at population-level are less useful in the context of a population dataset created through spatial microsimulation, even if the data are just simulated. In addition, in a dataset with several million observations, the *p*-values will nearly always be significant based on the relationship between power, effect sizes and sample size, independently of whether the (sometimes small) associations are meaningful in social research.^85^ Rather than statistical significance, the quantitative analyses therefore focus on beta coefficients as standardised effect sizes, which can be interpreted by comparison to other variables in the model. Model fit can be evaluated with conventional goodness-of-fit measures such as the adjusted R^2^, although based on the nature of the spatial microsimulation process and the way in which the outcome variables were constructed, the R^2^ values will be inflated, as a large share of the variance is explained by the constraint variables.

In addition, the data has a certain clustered structure with OAs and individuals within them. In general, there are two major ways of dealing with clustered data: multi-level models in which the variance is split between OAs and for individuals within them, and clustered standard errors.^86^ In this case, the latter may be more appropriate in the context of this research, as the outcome variables on the OA level are not really measured at the between level: perceived health and health service use are broken down by constraint variable, so that for perceived health, there are up to 700 possible values based on the five individual constraint variables and their categories per OA (each, on average, only has about 300 people). Therefore, the second option would be more appropriate, but adjusting standard errors without using *p*-values has no effect on the results. As a consequence, the data is analysed as a population dataset with a non-hierarchical structure.

Finally, it is worth discussing the benefits of using the spatial microsimulation approach, as opposed to simply appending the adjusted values for health status and health service use to OxIS. First, by including more than the 260 OAs contained in OxIS (2013), the values for perceived health and health service use from all 171,372 OAs in England can be included. This helps to randomise the error for the different outcome values for each OxIS individual, as some OxIS respondents are close to the mean of the assigned outcome values in their OA, whereas others substantially diverge from it. Of course, the analytical gold standard would be if census and HES data were not aggregated, but could actually be matched one-to-one to OxIS data; however, this is not possible due to good privacy/data protection-related reasons. In addition, from a practical point of view, the simulated estimates for all over England are more useful when deriving practical implications for specific areas within England, which would not be possible by using just the OxIS dataset.

## Results

As an initial step, the data can be explored with regressions to understand the general links between Internet use and health. A first analysis treating Internet use as the dichotomous question of use vs non-use confirms that Internet use and perceived health are positively related (β = 0.031), while Internet users use health services less frequently (β = −0.027, both not shown in Table 5), even after controlling for the known sociodemographic covariates that both influence Internet use and health.^43^

**Table 5.** Regression for Internet use, perceived health and health service use for access user concept (standardised/beta coefficients).

Independent variables	Perceived health	Health service use
Next-generation user	0.067	–0.037
First-generation user	0.034	–0.017
Ex-user	0.004	0.015
Never-user	(omitted)	(omitted)
Age	–0.207	0.433
Gender	0.094	0.015
Education	0.314	–0.031
NS-SeC	–0.083	0.033
Long-term condition	–0.462	0.030
**Adjusted R^2^**	**0.60**	**0.24**
***n***	**42,989,620**	**42,989,620**
**Largest condition index**	**6**	**6**

Note that outcome values for health service use were not adjusted for education, National Statistics Socio-Economic Classification (NS-SeC) and long-term health conditions due to the non-availability of these items in Hospital Episode Statistics (HES) data (see Methods section).

This also holds true when employing the more sophisticated concept of differences in access with next-generation users, first-generation users, ex-users and never-users.^36^ Table 5 shows that the positive relationship with perceived health is stronger for next-generation users than for first-generation users (β = 0.067 and 0.034), although this difference diminishes for health service use (β = −0.037 and −0.017, compared to never-users as the omitted category). The effect size for ex-users is very small for both (β = 0.004 and 0.015), indicating that ceasing to use the Internet is barely related to either outcome concept.

**Figure 3.**
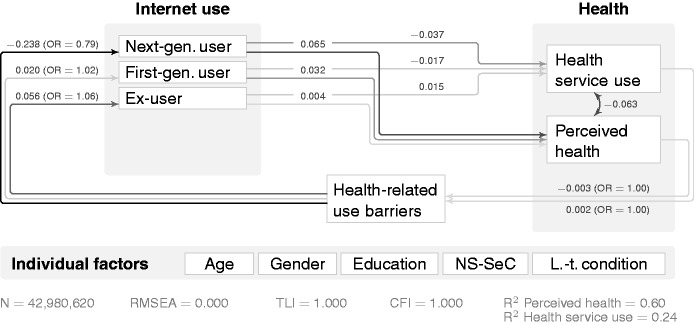
Structural equation modelling (SEM) for Internet use, perceived health and health service use for access user concept (standardised/beta coefficients). Paths have been omitted to improve clarity: from age, gender, education, National Statistics Socio-Economic Classification (NS-SeC), long-term condition to all variables in the model (next-generation user, first-generation user, ex-user, health service use, perceived health and health-related use barriers), as well as covariances between each of the Internet use concepts (next-generation user, first-generation user, ex-user). RMSEA: Root Mean Square Error of Approximation; TLI: Tucker-Lewis Index; CFI: Comparative Fit Index.

Structural equation modelling (SEM) helps to better understand the detailed mechanisms, especially the pathways in which one affects the other. Figure 3 provides insights into their potential causal links by incorporating the feedback loop with the OxIS survey concept ‘I have a health problem that limits my ability to use the Internet’. While the direct relationship between Internet use and perceived health remains strongest for next-generation users (after controlling for age, gender, education, NS-SeC and long-term conditions), next-generation use is least likely for those who agree that health limits their Internet use (β = −0.238).

The support user concept (Table 6) confirms the general differences between users and non-users, but leads to another interesting insight: for unsupported users, the association with perceived health is reduced almost to the level of non-users (β = 0.026), supporting the argument that those with unfulfilled support needs may not derive as much value from using the Internet.^38^ At the same time, perceived health and the reduction in health service use were highest for those who figured things out online without help (β = 0.071 and β = −0.061). For non-users, the relationship between both proxy access and proxy availability to perceived health is positive, but rather weak overall (|β| ≤ 0.016).

**Table 6.** Regression for Internet use, perceived health and health service use for support user concept (standardised/beta coefficients).

Independent variables	Perceived health	Health service use
Independent user	0.071	–0.061
Supported user	0.045	–0.030
Unsupported user	0.026	–0.023
Proxy access	0.016	–0.007
Proxy availability	0.009	–0.010
Fully excluded	(omitted)	(omitted)
Age	–0.218	0.437
Gender	0.092	0.150
Education	0.316	–0.031
NS-SeC	–0.084	0.032
Long-term condition	–0.462	0.030
**Adjusted R^2^**	**0.60**	**0.24**
***n***	**42,989,620**	**42,989,620**
**Largest condition index**	**8**	**8**

Note that outcome values for health service use were not adjusted for education, National Statistics Socio-Economic Classification (NS-SeC) and long-term health conditions due to the non-availability of these items in Hospital Episode Statistics (HES) data (see Methods section).

**Figure 4.**
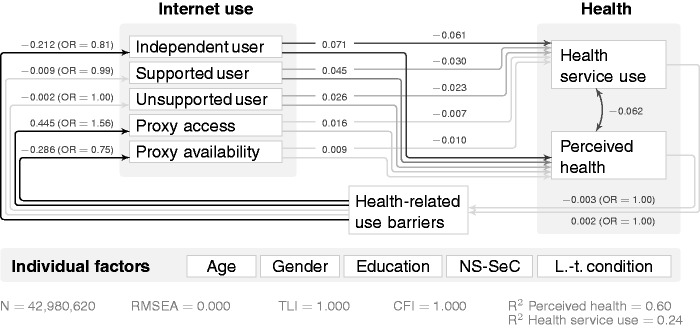
Structural equation modelling (SEM) model for Internet use, perceived health and health service use for support user concept (standardised/beta coefficients). Paths have been omitted to improve clarity (analogously to Figure 3). NS-SeC: National Statistics Socio-Economic Classification; RMSEA: Root Mean Square Error of Approximation; TLI: Tucker-Lewis Index; CFI: Comparative Fit Index.

Figure 4 shows that having a long-term condition that reduces the ability to use the Internet was connected to lower chances of independent use (β = −0.212). Interestingly, proxy access was more common among those who said that their health represented a barrier to use (β = 0.445), while they were less likely to belong to the group who had someone available without making use of this proxy (β = −0.286).

**Figure 5.**
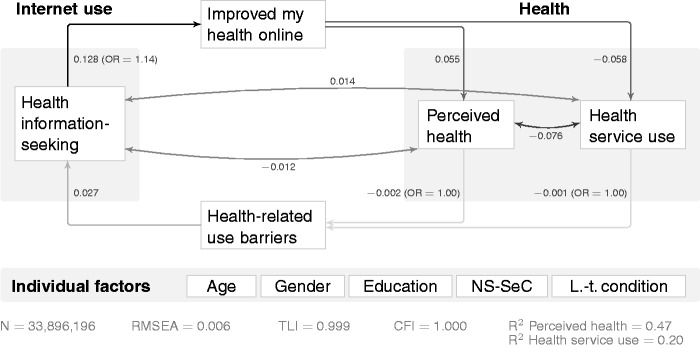
Structural equation modelling (SEM) model for Internet use, perceived health and health service use for usage user concept (standardised/beta coefficients). Paths have been omitted to improve clarity (analogously to Figures 3 and 4). NS-SeC: National Statistics Socio-Economic Classification; RMSEA: Root Mean Square Error of Approximation; TLI: Tucker-Lewis Index; CFI: Comparative Fit Index.

In terms of usage, health information seeking was barely related to perceived health (β = −0.005) and health service use (β = 0.009) while controlling for demographic covariates. However, incorporating the causally informed variable ‘Did you ever find anything online that helped improve your health?’ in Figure 5 shows an interesting relationship: using the Internet for health information seeking was strongly related to saying that the Internet helped improve one’s health (β = 0.128), which in turn shows a positive link to perceived health (β = 0.055) and is associated with lower levels of health service use (β = −0.058).

In further research based on the dataset generated in this research,^34,35,87,88^ a variety of other concepts from the quantitative data are analysed, and supplemented with qualitative insights in a mixed methods design. The qualitative data has been obtained through following up individuals from the original OxIS survey (as contact details could be obtained for those individuals who had agreed to being contacted again), in addition to further individuals from the same OAs used in OxIS. Qualitative follow-up data does not only serve to illustrate quantitative findings, but is also another way of validating the findings in the real world.

## Discussion

This article presented a way to simulate a dataset to research Internet use and health, validate the resulting dataset in itself and with alternative data sources, and enrich it with qualitative data. By connecting independent datasets, spatial microsimulation is an ‘innovative way of combining diverse datasets in order to understand health and wellbeing’ (p. 7) as set out in the Economic and Social Research Council (ESRC) long-term strategic priorities.^89^ At the same time, this research links to the priorities of the Medical Research Council (MRC) to better understand the complex relationship of lifestyle, inequalities and other measures from outside the healthcare setting.^90^ Through spatial microsimulation, aspects that would have traditionally been considered less relevant in health research can be included – both in relation to Internet use and the incorporation of other area-based characteristics – which may be important determinants of health.^43^

Various ways of validating the output, as one of the key stages in model building,^91^ showed that the dataset obtained through spatial microsimulation is a useful source for deriving new insights. While some error is introduced partially through IPF, integerisation and record swapping in the census data,^92^ internal validation showed that the differences between the actual and the simulated datasets are small. Testing how many individuals were replicated into their original areas showed that no individuals were systematically excluded, and a relatively high proportion were selected into their area. It is natural that this number does not reach 100% in any given simulated dataset due to the probabilistic nature of how individuals are selected by the TRS algorithm, particularly for more ‘interchangeable’ individuals (those who have more common characteristics in terms of the constraint variables). External validation with three independent data sources containing high-level questions on Internet use and health shows that the spatial microsimulation approach is an acceptable approximation of the real world, insofar as the surveys are a true representation thereof.

It is important to keep in mind the challenges related to using aggregate and individual data. This mainly refers to the ecological and the atomistic fallacies due to using aggregate data for making assumptions about individuals.^93^ However, compositional effects, such as OAs that featured a disproportionate number of one population group, are taken into account through simulating the dataset based on the total counts of the constraint variables per OA. In addition, assigning everyone the average for the specific group of gender, age, socio-economic status, education and long-term health conditions (as opposed to the average values per OA) allows a reasonable approximation of the individual measures, and avoids the bias of assigning the same value to the 60-year-old man of low socio-economic status and with a long-term condition as to the healthy 25-year-old woman of high socio-economic status just because they are from the same area, as is usually a problem with ecological inference.^81^ Of course, the relative socio-economic homogeneity within one OA, which has been specifically accounted for in the design of the OAs in the census, is an important prerequisite for this analysis.

The results showed that there is some relationship between Internet use and both perceived health and health service use, with higher levels of perceived health and lower levels of health service use independently of whether Internet use was conceptualised in terms of access, support or usage. Of course, as the SEM analyses indicated, part of the relationship may be explained by health affecting Internet use, as health problems may keep individuals from using the Internet on certain devices,^36^ increase the odds of being a proxy user,^1^ and trigger health information seeking online more generally.

Then again, part of the relationship with perceived health may be explained by Internet use affecting health. This confirms previous research^7,9,14^ even though, in some cases, Internet use may also lead to lower levels of perceived health, as captured by the lifestyle paradox.^87^ In line with previous research, Internet use was found to reduce health service use.^7,10^ Interestingly, in contrast to the results presented in this paper, other research also found that health service use was increased due to using the Internet,^11–13^ which may be attributed to looking at health information seeking (as opposed to health information finding or Internet use general), and potentially not controlling properly for individuals’ long-term health conditions – while an overall negative relationship as found in this research does not exclude a positive relationship for some individuals.^35^

Accessing the Internet on multiple, mobile devices (‘next-generation users’) is more strongly related to both outcome concepts, although there is also a positive relationship for first-generation users. Part of this may be explained by next-generation users being more likely to see the Internet as a convenient first port of call, while also creating content and using the Internet to interact with other people more frequently,^36^ given that socioeconomic and educational differences between next-generation and first-generation users were controlled for. At the same time, the results suggests differences between supported and unsupported users particularly for perceived health, with unsupported users being close to the level of proxy users. This shows the importance of the social environment,^38^ both for help and as a general source of support,^94^ which may explain why there is a weak relationship to perceived health and health service use even for those who only have a proxy available (without having asked them to use the Internet). Finally, what individuals do online and whether they find what they are looking for matters:^40,41^ while health information seeking itself did not have any relationship with either outcome concepts, finding something online was related to higher levels of perceived health and lower levels of health service use. This may again point to the importance of online skills, for example for finding and evaluating health information online.

## Conclusion and outlook

Based on the simulated dataset created in this research, Internet use is related to higher levels of perceived health and lower levels of health service use independently of whether Internet use was conceptualised in terms of how individuals access the Internet, how they are supported and are in need of support, and whether they use the Internet for health-related purposes (while controlling for the known sociodemographic influence factors on Internet use and health).

These insights may inform both theory and practice. On the theoretical side, this research shows how the digital divide literature helps to conceptualise Internet use, and how access, support and usage of the Internet are important in the context of examining associations with perceived health and health service use – with some level of reverse relationship shown by incorporating health-related barriers to Internet use and Internet-enabled effects on health and healthcare in the analysis. In addition, while existing theories on social support^32^ or building up social and information capital online^25,26^ may be applicable, little is known about the mechanisms behind the relationship between Internet use and health. To this end, pathways identified from SEM help to elicit mediating mechanisms by which Internet use influences health and the other way around.^35^

On the practical side, this research shows the positive relationship between Internet use and health, and the negative association with health service use. This is a valuable insight for policy, for example in the context of the current NHS ‘Widening Digital Participation’ programme,^22^ but also for the overall digital strategy across a wide range of countries,^23^ Further research based on the generated dataset provides insights for practitioners with respect to how Internet use influences patients’ decisions, and how practitioners may react and help with this process, especially how to support the positive effects of Internet use while reducing potentially adverse implications.^88^ In addition, further research based on the simulated dataset also looks at divergences from the identified relationship between Internet use, perceived health and health service use, for example in the context of the lifestyle paradox.^87^

While spatial microsimulation has already been proven useful in other health-related contexts,^44–49^ this research demonstrated a novel application of spatial microsimulation beyond estimating the geographical distribution of a certain phenomenon. Spatial microsimulation allows for linking datasets using geographical location as a connecting element, thereby providing a way to generate new insights from secondary data with no additional burden on participants (except for potential qualitative follow-up interviews to add value by enriching the simulated quantitative findings). With more and more data being generated in academic research and from routine sources within and outside health and social care systems, spatial microsimulation may gain importance as a privacy-considerate and cost-effective way of doing research.
